# Posterior reversible encephalopathy syndrome (PRES) in an HIV-1 infected patient with disseminated varicella zoster virus: a case report

**DOI:** 10.1186/1471-2334-13-396

**Published:** 2013-08-28

**Authors:** Sarah C Sasson, Aileen Oon, Joga Chagantri, Bruce J Brew, Andrew Carr

**Affiliations:** 1 HIV, Immunology and Infectious Diseases Unit, St Vincent’s Hospital, 390 Victoria Street Darlinghurst, 2010, Sydney, NSW, Australia; 2 The Kirby Institute, University of New South Wales, Sydney, Australia; 3 Department of Radiology, St Vincent’s Hospital, Sydney, Australia; 4 Department of Neurology, St Vincent’s Hospital, Sydney, Australia

**Keywords:** PRES, HIV, VZV, MRI, Encephalopathy, Vasculopathy

## Abstract

**Background:**

Posterior reversible encephalopathy syndrome (PRES) is an uncommon pathology characterized by the acute onset of headache, vomiting, altered consciousness, seizures and focal neurological deficits. It was initially described in the setting of hypertension, uremia and immunosuppression. In the last decade there have been emerging reports of PRES in patients with advanced human immunodeficiency virus (HIV)-infection in the presence of hypertension, dialysis, hypercalcaemia and two opportunistic infections: blastomycosis and tuberculosis (TB).

**Case presentation:**

Here we present the case of a 54 year old male being treated for disseminated varicella zoster virus (VZV) and vasculopathy in the setting of HIV infection who acutely deteriorated to the point of requiring intubation. His clinicoradiological diagnosis was of PRES and he subsequently improved within 72 h with supportive management. Serial neuroimaging correlated with the clinical findings. The pathogenesis of PRES is poorly understood but is thought to stem from vasogenic oedema either as a result of loss of endothelial integrity and transudate of fluid across the blood–brain barrier, or secondary to vasospasm resulting in tissue oedema in the absence of infarction. How HIV infection impacts on this model is unclear. It is possible the HIV infection causes endothelial dysfunction and disruption of the blood–brain barrier that may be further exacerbated by infections in the central nervous system.

**Conclusion:**

The phenomenon of PRES in advanced HIV is an important clinical entity for both physicians and critical care doctors to recognize firstly given its potential mortality but also because of its favourable prognosis and reversibility with supportive care and treatment of underlying causes.

## Background

PRES is a clinicoradiological entity characterized by the development of headache, hypertension, altered mentation, focal neurological deficits and seizures and by the MRI findings of symmetric and bilateral subcortical oedema including mainly the parietal and occipital lobes. Importantly the changes are transient and reversible. PRES was originally described in association with hypertension, uremia and immunosuppression [1]. The pathogenesis of PRES is poorly understood but is thought to involve vasogenic dysregulation and/or disruption of the blood–brain barrier. Current hypotheses include severe hypertension causing a failure of autoregulation with endothelial injury leading to vasogenic oedema. The alternate hypothesis is of vasoconstriction and hypoperfusion leading to brain ischemia and subsequent oedema in the absence of infarction. The typical anatomy of PRES, in the posterior regions, is thought to reflect poorer sympathetic innervation as compared to the anterior circulation.

There have been emerging reports that several pathogenic processes can lead to PRES in HIV-infected adults. In nine previously published cases, four were associated with hypertension [2,3], including two with end-stage kidney disease requiring dialysis [4,5], one with hypercalcemia [6], and two with disseminated infections (blastomycosis [7] and TB [8]). In one report no precipitating factor could be found [9]. While hypertension and endothelial damage/dysfunction appear to be two main factors contributing to PRES, HIV infection is also known to be associated with vascular changes, altered vascular reactivity and focal blood–brain barrier disruption [10]. Here we report the development of PRES in a patient with disseminated VZV with vasculopathy and advanced HIV infection that was severe enough to warrant intubation.

## Case presentation

A 54 year old male presented to another hospital with a three day history of headache, vomiting, diplopia and photophobia. His past medical history included advanced HIV infection (recent CD4 T-cell count 173 cells/μL and HIV Viral load 36 copies/mL), anal squamous cell carcinoma (SCC) treated with chemo- and radiotherapy and known single metastasis to the liver, and back pain. Medications at admission were: darunavir 600 mg PO BD, ritonavir 100 mg PO BD, etravirine 200 mg PO BD, raltegravir 400 mg PO BD esomeprazole 20 mg PO daily, paracetamol 1 g PO QID and oxycontin 20 mg PO BD. The patient lived independently and had a 40 pack-year history of smoking. On examination he was febrile to 38.2°C. All other vital signs were within normal limits. Cardiopulmonary and abdominal exams were normal. Neurologically he had an expressive dysphasia, a left sixth nerve palsy and mild global ataxia. Computer-tomography (CT) imaging of the brain was within normal limits. The patient underwent a lumbar puncture that showed 101×10^6^ leukocytes (95% mononuclear cells and 5% polymorphs), 20×10^6^ erythrocytes, elevated protein 3.44 g/L, and normal glucose 3.8 mmol/L. He was admitted with presumed meningitis and treated with ceftriaxone 2 g IV BD, benzyl penicillin 2.4 g IV q4h, dexamethasone 10 mg PO QID. Later on Day 0 he underwent a magnetic resonance imaging (MRI) scan of the brain that showed multiple non-enhancing lesions demonstrated on the T2 weighted sequences that were not associated with vasogenic oedema. These were considered most consistent with an infective cause and were not typical of metastatic disease. The patient was commenced on clindamycin 600 mg IV QID and pyrimethanine 25 mg/folinic acid for empirical treatment of toxoplasmosis. On Day 1 the patient developed a vesicular rash over his face, trunk and back. A vesicle was de-roofed and swabbed and the patient was commenced on aciclovir 700 mg IV TDS for presumed disseminated VZV prior to transfer to our centre.

**Figure 1:**
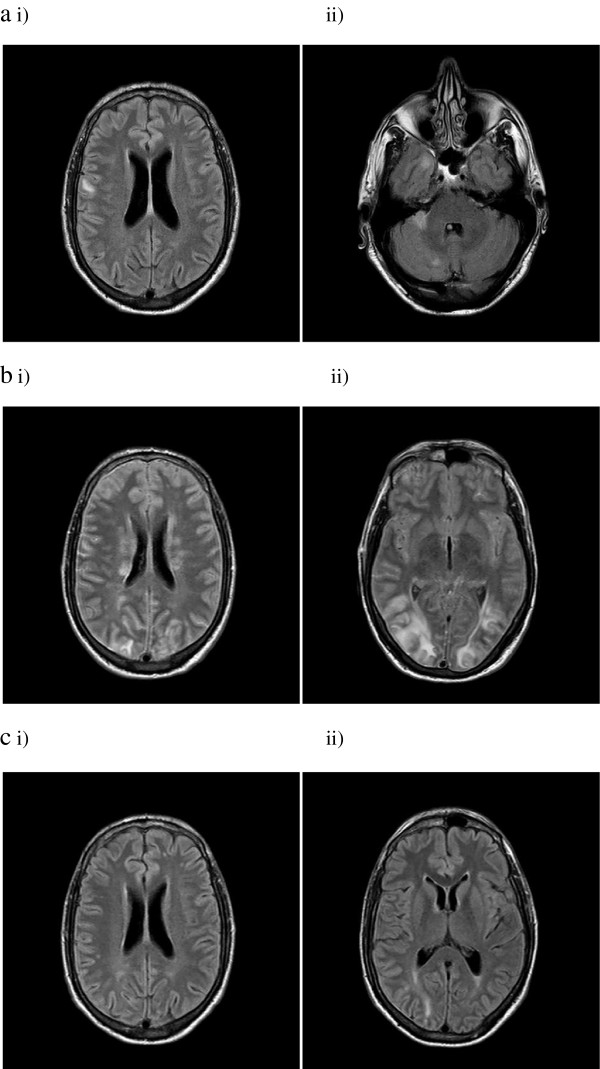
**Sequential T2- weighted FLAIR MRI Brain imaging. a)** Nodularly enhancing lesions on initial presentation in the i) right anterior temporal cortex and ii) right cerebellum. Subcortical U-fibre high signal intensity lesion in the right frontoparietal area as shown by T2- weighted FLAIR MRI. **b)** Marked interval increase in the signal hyperintensities demonstrated by T2- weighted FLAIR MRI in i) frontal, parietal and temporal lobes and the ii) cortical and subcortical/deep white matter of the occipital lobes following acute deterioration and consistent with PRES. **c)** Interval reduction of signal hyperintensities as shown by T2- weighted FLAIR MRI in i) parietal and ii) occipital lobes demonstrating resolving vasogenic oedema.

Upon arrival at our unit ceftriaxone and benzyl penicillin were ceased given the lack of supporting evidence for bacterial meningitis and dexamethasone reduced to 4 mg PO BD. A repeat MRI with T2 weighted FLAIR sequence showed nodularly enhancing lesions in the right cerebellum, right cerebellopontine angle and right anterior temporal cortex at the grey-white matter junction and a subcortical U-fibre high signal intensity lesion in the right frontoparietal area reported as possibly representing progressive multifocal leukoencephalopathy (PML) (Figure 1ai) and ii)). The swab from the vesicular rash was positive for VZV and our provisional diagnosis was disseminated VZV with associated cerebral vasculopathy. We planned for ten days of IV aciclovir with follow-up MRI and brain biopsy if the lesions had not resolved. Differential diagnoses included Toxoplasma, lymphoma and PML. Subsequently PCR detected VZV and Epstein-Barr virus (EBV) in the CSF but testing for Cryptococcal antigen and JC virus were negative.

On Day 10 the patient complained of severe bilateral headache. He was afebrile with a blood pressure (BP) of 165/62. There were no new focal neurological deficits and he was treated with 10 mg PO oxycodone. Three hours later he suffered an unwitnessed fall. BP at this time was 170/96. An additional three hours later he had a witnessed generalized tonic-clonic seizure (GTCS) associated with tongue biting and loss of urinary and fecal continence. His BP was 170/90. He received diazepam 20 mg IV and was loaded with phenytoin 1 g IV. Eight hours following this he had a second witnessed GTCS associated with fever to 39.3°C. His oxygen saturation was 98% on room air and BP was 131/82. On review he would not open his eyes, made no sounds and extended to painful stimuli with global hyper-reflexia and a left Babinski sign. The patient underwent immediate ICU review for airway management to facilitate an urgent CT Brain. The CT showed hypoattenuating areas in the right frontal lobe and bilateral occipital lobes suggesting acute PRES. A subsequent MRI with T2 weighted FLAIR sequence was also consistent with PRES (Figure 1bi-ii).

The patient was taken to ICU for intubation. His phenytoin was switched to levetiracetam 500 mg IV BD due to deranged liver function tests. The consultant neurologist made a provisional diagnosis of PRES secondary to progressive VZV vasculopathy. Serum creatinine (91 μmol/L; eGFR 75) and calcium levels (2.44 mmol/L) were within normal limits. The differential diagnoses included immune-reconstitution-inflammatory syndrome (IRIS) and PML (although the JC virus PCR from CSF was negative). The radiological findings were not consistent with toxoplasmosis. An electroencephalogram did not show epileptiform activity. Three days after intubation the patient was hypertensive to 172/88 with a mean arterial pressure (MAP) consistently over 100 (in the 3 days prior his MAP was stable at 80). He was treated with IV hydralazine PRN before being commenced on amlodipine 10 mg via a nasogastic (NG) tube and captopril 25 mg NG TDS. His antiretroviral therapy (ART) treatment was withheld for three days due to the incompatibility of some drugs with NG administration. Two days after intubation the patient’s sedation was weaned and he was able to follow commands. He was extubated three days after intubation at which time his eyes opened spontaneously, he was speaking spontaneously and obeying commands, and was returned to the ward. A follow-up MRI showed resolution of vasogenic oedema on T2 weighted FLAIR sequence (Figure 1ci-ii). A CT angiogram did not find evidence of a vasculopathy at this stage. The patient completed 14 days of IV aciclovir, his dexamethasone was weaned and ceased and clindamycin, pyrimethanine, folinic acid and captopril ceased. After a week in an inpatient rehabilitation unit he was discharged home with a persistent left sixth nerve palsy.

## Conclusions

To our knowledge this is the first report of PRES in an HIV-infected patient in the setting of disseminated VZV and associated vasculopathy. Additionally it is the first reported case of HIV-associated PRES severe enough to warrant intubation. Here, the presentation of a patient with fevers, headache and visual disturbance and subsequent findings of MRI lesions at the grey-white matter junction, CSF containing a mixed mononuclear leukocytosis and erythrocytosis and VZV IgG are all consistent with the diagnosis of VZV vasculopathy [11]. This condition results from productive viral infection of cerebral arteries and has a range of known sequelae including aneurysm, cerebral and subarachnoid hemmorrhage, arterial ectasia and carotid artery dissection [11]. VZV vasculopathy is generally treated with IV acyclovir with or without concomitant steroids. It is thought that steroids may reduce associated inflammation, however this should be weighed against the risk of promoting viral infection [11]. Overall the evidence for steroid use in this condition remains unclear [12]. To our knowledge this is the first report of VZV vasculopathy associated with PRES, and it is likely this syndrome is part of the ever-widening spectrum of VZV-associated vascular disease.

In previously published cases of HIV-associated PRES, all had similarly advanced immunodeficiency with seven of nine patients having CD4 counts <200 cells/μL [2-8] while the remaining were <300 cells/μL [3,9]. Interestingly, here the patient had no history of hypertension and throughout the first 11 days of admission his BP had a baseline of 140/90. The highest recorded BP was 172/88 triggering treatment. Possible risk factors contributing to endothelial damage in this patient include: undiagnosed hypertension, smoking, HIV infection [13] and the use of protease inhibitors [14]. This case is similar to that reported by Saeed *et al.*[7] of an HIV-infected patient with no previous hypertension presenting with a disseminated infection and developing concurrent hypertension during admission.

Although rare, PRES is important to recognize in order to reduce associated morbidity and mortality. It is now becoming apparent that patients with advanced HIV infection and CD4 counts <300 cells/μL may be at risk of developing PRES either in isolation or in the setting of hypertension, hypercalcemia or disseminated opportunistic infection, particularly those penetrating the CNS. Fortunately prognosis with supportive care and reversal of underlying causes is good.

## Consent

Written informed consent was obtained from the patient for publication of this case report and any accompanying images. A copy of the written consent is available for review by the Editor of this journal.

## Abbreviations

ART: Antiretroviral therapy; BD: Twice daily; BP: Blood pressure; CNS: Central nervous system; CSF: Cerebrospinal fluid; CT: Computer-tomography; EBV: Epstein-Barr virus; eGFR: Estimated glomerular filtration rate; GTCS: Generalized tonic-clonic seizure; HIV: Human immunodeficiency virus; ICU: Intensive care unit; IRIS: Immune-reconstitution-inflammatory syndrome; IV: Intravenous; MAP: Mean arterial pressure; MRI: Magnetic resonance imaging; NG: Nasogastic; PCR: Polymerase chain reaction; PML: Progressive multifocal leukoencephalopathy; PO: Per os; PRES: Posterior reversible encephalopathy syndrome; PRN: Pro re nata; QID: Four times a day; SCC: Squamous cell carcinoma; TB: Tuberculosis; TDS: Three times daily; VZV: Varicella zoster virus.

## Competing interests

Financial disclosures: SS, AO and JC: Nil financial disclosures. BB has received research funding from Eli Lily, Glaxo Smith Kline, ViiV Healthcare and Merk Serono; Travel sponsorship from Abbott; Honouraria from ViiV Healthcare, Boeringer Ingleheim, Abbott, Biogen Idec and Abbvie and has served on Scientific Advisory Boards for Glaxo Smith Kline, Biogen Idec, ViiV Healthcare and Merk Serono. AC has received research funding from Baxter, Gilead Sciences, MSD and Pfizer; consultancy fees from Gilead Sciences, MSD, and ViiV Healthcare; lecture and travel sponsorships from Gilead Sciences, MSD, and ViiV Healthcare and has served on advisory boards for Gilead Sciences, MSD, and ViiV Healthcare. The authors declare that they have no competing interests.

## Authors’ contributions

SS cared for the patient, ordered and interpreted diagnostic tests, performed the literature review and wrote the manuscript. AO cared for the patient, ordered and interpreted diagnostic tests and reviewed the manuscript. JC interpreted and reported radiological tests and reviewed the manuscript. BB cared for the patient, interpreted diagnostic tests and provided significant edits to the manuscript. AC cared for the patient, interpreted diagnostic tests and provided significant edits to the manuscript. All authors read and approved the final manuscript.

## Pre-publication history

The pre-publication history for this paper can be accessed here:

http://www.biomedcentral.com/1471-2334/13/396/prepub
